# A 2-Year Randomized Clinical Trial of Three Bonding Techniques in Non-Carious Cervical Lesions

**DOI:** 10.3390/medicina60061005

**Published:** 2024-06-19

**Authors:** Eftychia Pappa, Grigoria Gkavela, Ioanna Sampri, Konstantinos Masouras, Christos Rahiotis, Afrodite Kakaboura

**Affiliations:** Department of Operative Dentistry, Dental School, National and Kapodistrian University of Athens, 11527 Athens, Greece; effiepappa@dent.uoa.gr (E.P.); grigoriagk@dent.uoa.gr (G.G.); giannaas@hotmail.com (I.S.); kmasoura@dent.uoa.gr (K.M.); akakabou@dent.uoa.gr (A.K.)

**Keywords:** self-etch, total-etch, selective enamel etching, retention, caries, marginal adaptation, marginal staining, non-caries cervical lesions

## Abstract

*Background and objective*: The aim of this randomized split-mouth study-controlled clinical trial was to compare the 2-year clinical performance of resin composite restorations placed at non-caries cervical lesions (NCCL) with one-step self-etch, total-etch, and selective enamel etch and self-etch adhesive techniques. *Materials and methods*: Thirty-two patients received three resin composite restorations each at NCCLs (Tetric EvoCeram/Ivoclar/Vivadent), bonded with a total-etch adhesive agent (ExciTE F/Ivoclar/Vivadent) and a self-etch (AdheSE One F/Ivoclar/Vivadent) without and with selective enamel etching. All restorations were evaluated by two examiners at baseline, 6-, 12-, 18-, and 24-months with FDI clinical criteria (post-operation regarding retention, caries occurrence, marginal adaptation, and marginal staining). A logistic regression analysis, a Cohen’s kappa statistic, a multifactorial analysis, and X^2^ were performed with generalized estimating equations. *Results*: After 2 years, the retention rate was 86.8% for total etch, 92.26% for self-etch, and 93.63% for selective enamel etching and self-etch. No caries was detected on the restorations. Concerning marginal adaptation, the clinically perfect restorations were 26.9% for the total-etch technique, 16% for self-etch, and 25.9% for selective enamel etch and self-etch. The logistic regression model revealed that only time reduced the probability of perfect marginal adaptation. *Conclusions*: All three adhesive strategies provided restorations with no significant differences in the retention rate or marginal adaptation, whereas the total etch yielded better performance for marginal staining. All restorations were assessed as clinically acceptable after 2 years.

## 1. Introduction

One-step self-etch adhesives are the latest evolution in adhesive systems for resin composite restorations. The rationale for introducing self-etch adhesives was to simplify and accelerate the application technique. By integrating the etching step into the bonding procedure, it was presumed that all the potential over-etching disadvantages [1] and humidity-related limitations [1] observed with the etch-and-rinse (total-etch) adhesives could be surpassed. Moreover, the retention of the smear layer could prevent tooth sensitivity [2].

Unfortunately, not all self-etch adhesives effectively etch enamel [3]. Thus, selective enamel etching with phosphoric acid was proposed in conjunction with the use of self-etch adhesives [3,4,5,6]. In addition, many studies showed inferior bonding performance and durability of one-step self-etch agents compared with etch-and-rinse and two-step self-etch adhesives [7,8].

Laboratory testing of bonding systems has already provided sufficient data about their features under in vitro conditions [7]. Nevertheless, since the oral environment differs considerably, experimental testing cannot always project clinical performance in a predictable manner. Thus, clinical trials are needed to identify the behavior of the adhesive systems. As a function of time, further outcomes were achieved by more clinical studies conducted [1]. However, it is notable that remarkable differences regarding the findings are revealed among the investigations, even in trials that evaluate the same agents [9,10,11,12,13].

Non-carious cervical lesions (NCCL) are characterized by a loss of hard dental tissue near the cement-enamel junction. Commonly, their shape is like a wedge, with the apex pointing inward. Other times, they appear as regular depressions, like a dome or a cup. Their etiology seems to be related to extrinsic and intrinsic acids, mechanical abrasive forces, and tooth flexure. NCCLs have been extensively used in the clinical testing of bonding systems due to their significant advantages [9,10,11,12,13,14]. Firstly, many NCCLs commonly exist in a mouth, providing sites for different restoration techniques that can be subsequently evaluated under similar conditions. Secondly, their shape does not provide any undercuts, which could favor any mechanical retention of the restoration. Also, at NCCL, the enamel margins are usually limited in length and are thin compared with other restorations, so the adhesion reflects mainly the ability to adhere to dentine, which is considered the weak part regarding the bonding [15]. Apart from the clinical efficacy of the bonding techniques, this study additionally correlates different individual parameters influencing the clinical characteristics of restorations.

The primary aim of this randomized split-mouth controlled clinical trial was to compare the 2-year clinical performance of resin composite restorations placed at NCCLs with one-step self-etch, total-etch, selective enamel etch, and self-etch adhesive techniques. The null hypothesis tested was that there is no difference among these three bonding techniques regarding the evaluated clinical characteristics.

## 2. Materials and Methods

The clinical trial was a split-mouth, single-center, randomized, controlled study with blinding patients and clinical evaluators’. Before patient enrollment, the Committee for Research and Ethics of the School of Dentistry, National and Kapodistrian University of Athens, Athens, Greece, approved the research protocol (Register No. 118). The clinical trial conforms to the guidance set by ClinicalTrials.gov (NCT04565938).

The inclusion criteria for patients to participate in the study were: (a) being 19 years of age or older; (b) having good general health; (c) being available for follow-up visits; and (d) having at least 20 teeth. The exclusion criteria were: (a) rampant uncontrolled caries/high caries activity; (b) advanced untreated periodontal disease or receiving periodontal treatment; (c) >2 cigarette packs/day or equivalent chewing tobacco; (d) systemic or local disorders that contra-indicate dental procedures included in this study; (e) evidence of xerostomia; (f) evidence of severe bruxism, clenching, or TMD; and (g) patients undergoing orthodontic treatment.

The selection pool consisted of patients receiving dental treatment in the postgraduate clinic of restorative dentistry. Two calibrated Operative Dentistry residents selected 32 participants who met the inclusion criteria. Before treatment, written informed consent was obtained from all participants.

Patients, irrespective of age and gender, had at least three NCCLs on the incisors and/or canines and/or premolars of the upper or lower jaw. Each lesion was located at the cervical third of the buccal surface of the tooth, either at the same level or above the gums, with the cervical wall placed on dentin, not extending on adjacent surfaces, and not exceeding 5 mm in length, 3 mm in height, and 1.5 mm in depth. A total of ninety-six lesions were eventually employed in thirty-two patients.

Each patient answered questions related to their age, frequency of tooth brushing, and dental visits. In addition, the number of teeth with abraded, non-carious cervical surfaces was recorded.

The restorations were performed by a single experienced dentist (I.S). All three lesions per patient were restored with Tetric EvoCeram (Ivoclar/Vivadent, AG, Shaan, Lichtenstein) (Table 1) following one of the subsequent three adhesive procedures for each tooth, in a statistically random order using randomization tables. The first randomly selected method was used to restore the tooth with the lowest tooth number (according to the FDI system), and the second method was used for the tooth with the second lowest number and the third one with the highest. The randomization was performed by a person not involved in the study.

All the cavities restored required mechanical removal of sclerotic dentin with a round diamond bur and enamel beveling (0.05 mm wide). Then, each of the three teeth per patient received one of the three adhesive procedures tested. The materials used and the application procedure per adhesive agent are presented in Table 1. The bonding techniques were applied according to the manufacturer’s instructions.

Method A: enamel was etched with 37% phosphoric acid for 30 s and dentin for 15 s, ExciTE F adhesive agent was applied and photocured with 800 mW/cm^2^ light intensity (Cure TC-01, Spring Health Products, Inc., Norristown, PA, USA), resin composite Tetric EvoCeram was placed, a transparent cervical matrix (Kerr-Hawe, Orange, CA, USA) covered its surface, the resin composite was photopolymerized for 40 s, and finishing/polishing was followed with diamond finishing burs, polishing disks, and silicone polishers.

Method B: enamel etching was not performed; AdheSE One F adhesive agent was applied, and the next clinical steps were the same as in method A.

Method C: enamel was etched with N-etch for 30 s, then AdheSE One F adhesive agent was applied, and the next clinical steps were the same as in method A.

Two experienced and calibrated examiners performed the evaluation. The examiners examined and scored 10 restorations in a pilot study, different from those included in the study. Before starting the evaluation, an intra-examiner and inter-examiner agreement of at least 85% was necessary. These examiners were not involved with the restoration placement, and therefore, they were blinded relative to the group assignment. The patients did not know which teeth had which restoration. All restorations were evaluated at baseline, 6-, 12-, 18-, and 24-months post-operation, based on the FDI clinical criteria introduced by Hickel et al. 2010 [16]. Upon completion of the restorations, all participants were instructed to use the brass technique of brushing.

The parameters evaluated were retention of the restoration, occurrence of caries, marginal adaptation, and marginal staining. The primary outcomes were retention and the presence of caries. The secondary was the marginal adaptation and staining. Retention and occurrence of caries along the restoration margins were scored as yes or no. The criteria for marginal adaptation and staining were graded from 1 to 5 according to the description suggested by Hickel et al. [16].

### Statistical Analysis

The study’s sample size was calculated using G*Power 3.1 software. This study used two methods to estimate the sample size. The first was for the multiple regression analysis. We used the parameters of seven independent variables (time, bonding technique, age, gender, frequency of brushing, visit to the dentist, and number of teeth with abraded cervical surfaces), α = 0.05, β = 0.8, and a considerable effect size measure for F2 0.35. The minimal sample size was estimated at 26 patients. The second method for comparing the 3 methods is the survival rate. A trial design was considered, with a percentage of success at 90% among the treatments and an ability to detect differences greater than 20% [17]. The number of restorations was 25 per group, based on an 80% power and a statistical significance level set at 0.05. This study included 32 patients to balance a possible drop-out.

Absolute (number of observations) and relative (percentage ratios) frequencies were used to describe the qualitative characteristics of the present study. The chi-square and Mann-Whitney tests were used to compare the qualitative characteristics. The description of the quantitative characteristics was based on calculating the interquartile range. It is stated that the statistical significance in cases where there was more than one observation was based on the data of an observation of each individual, which was selected randomly [18]. This was done as there is expected to be a correlation between the repeated observations for the same person, and thus, the essential condition of the independence of the observations does not apply. However, the calculation of the other statistical significances was based on appropriate models considering the correlation between the observations of the same individual.

A logistic regression analysis with generalized estimating equations accounted for the clustered data (three restorations per patient, backward selection method). Cohen’s kappa statistic was used to test inter-examiner agreement for the four evaluated parameters. Estimates from appropriate random effects and logistic regression models were used to capture the likelihood of the course of clinical features assessed over time (random effects and logistic regression). To calculate the respective 95% confidence intervals (95% CI), it was necessary to calculate the standard errors (SE) of the estimates based on the multivariate delta method.

The probability of formation in the control period of the clinical criteria—which presented a deviation from perfect—per technique was estimated using the non-parametric Kaplan-Meier survival test, while the control of the differentiation of these percentages among the three techniques, overall, at all times, was based on the non-parametric log-rank test.

Also, a multifactorial analysis was performed. The variables time, bonding technique, age, gender, frequency of brushing, visit to the dentist, and number of teeth with abraded cervical surfaces were included in the final models regardless of the degree of statistical significance. The level of significance was set at *p* < 0.05. Based on the models mentioned, the effect of the restorative technique on the probability of the course of each clinical criterion over time was estimated, which was corrected for the possible effects of other potential confounders.

All tests were performed with SPSS (IBM, Ver 16.0) statistical software.

## 3. Results

The distribution of the lesions per method is presented in Table 2.

**Figure 1:**
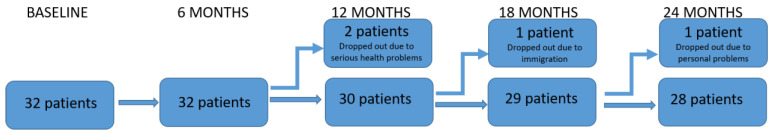
Flowchart of the participants along the study, indicating losses in follow-up and reasons.

The majority of thirty-two patients were women (59.4%), and the average age of the patients was 59.5 years. The recall rate at 6 months was 100%, whereas after 24 months, the overall recall rate was 87.5% (Figure 1). The descriptive statistics of patient-related parameters are presented in Table 3. No statistically significant differences were detected in the characteristics of the patients in relation to their gender.

Table 3, Table 4 and Table 5 presented the retention rate, the percentage of marginal adaptation, and marginal discoloration at different time intervals. No statistically significant differences were revealed among the three bonding techniques in terms of retention rates for all the recalls (Table 4).

No caries were observed at any restoration or evaluation time.

Statistical analysis proved no significant differences between the bonding methods for all follow-up times and grades regarding marginal adaptation (Table 5).

No statistically significant differences were detected among the bonding methods for all follow-up times and grades concerning the marginal staining (Table 6).

Regarding the probability of correlation of the perfect marginal adaptation, in all observation times, with the parameters of time, age, gender, bonding method, frequency of brushing, visit to the dentist, and number of teeth with abraded cervical surfaces as independents, only time had a statistically significant effect. The probability of a perfect marginal adaptation decreased, and the relative decrease was estimated to be 17.9% per month (*p*-value <0.001).

As far as marginal staining, over time, the probability of absence of marginal staining decreased, and the relative decrease was estimated to be 11.8% per month (*p*-value < 0.001). The reduced probability of absence of marginal staining by 88.9% (relative difference) compared with the total-etch one (*p*-value < 0.001) was calculated with the self-etch method. Accordingly, the technique with selective enamel etch and self-etch had a reduced chance of no straining by 84.5% (relative difference) compared with the total-etch one (*p*-value < 0.001).

**Figure 2:**
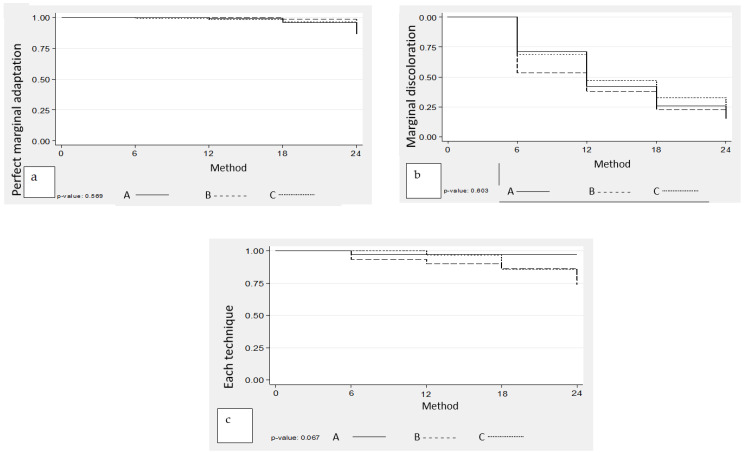
The Kaplan-Mayer graphs of the percentage of retention rates (**a**), of perfect marginal adaptation (**b**), and marginal discoloration (**c**) for each technique throughout the control period.

Figure 2 presents the Kaplan-Mayer graphs of the percentage of retention rates of perfect marginal adaptation and marginal discoloration for each technique throughout the control period.

The percentage of retention, perfect marginal adaptation, and absence of marginal discoloration do not show statistically significant differences among the three methods.

Table 7 presents the results of the logistic regression analysis of the probability of correlation of the perfect marginal fit with all the abovementioned parameters.

In the univariate and multivariate logistic regression models, it is observed that, of all parameters, only time has a statistically significant effect, reducing the probability of perfect marginal adaptation (estimated relative reduction per month: 17.9% in the univariate model and 18.2% in the multivariate model, respectively). In the univariate analysis that did not correct for time, the values of the estimated effect of the technique on the likelihood of marginal adaptation were found to be smaller. The logistic regression analysis shows that none of the independent variables (gender, age, frequency of brushing and visiting the dentist, number of teeth with abraded teeth, shape of damage) influence the probability of perfect marginal adaptation over time.

The Cohen’s kappa value between examiners to test inter-examiner agreement for each of the four evaluated parameters is almost perfect (over 0.91).

## 4. Discussion

The research and development of dental adhesives have mostly focused on making the clinical procedure more user-friendly by reducing the number of bottles and/or application steps. Clinicians certainly prefer less complicated and more versatile adhesive materials. However, a trade-off exists between simplifying dental adhesives and clinical outcomes [7]. The current study investigated the clinical performance of a self-etch, a total-etch, and a selective enamel etch bonding technique employed to restore non-caries cervical cavities after 24 months of oral function.

Thirty-two patients enrolled in the trial, with a mean age of 59.5 years, the majority of whom were women. At 6 months, the re-examination rate was 100%, and at 24 months, it reached 87.5%. The reasons for drop-off were the inability of two patients to participate in the 12-, 18-, and 24-month reviews for serious health reasons, of one patient at 18- and 24-months due to immigration, and of one patient at 24 months due to personal problems.

Apart from the favorable sample size and the study’s power, this clinical trial was adequately randomized and exhibited a double-blinded evaluation. A single resin composite material was used with all bonding procedures to rule out the adhesives’ composite-related influence on the restorations’ performance.

Before the restorations were placed, we removed the superficial layer of the sclerotic dentin by grinding. Sclerotic dentin lesions show dentinal tubule formation that is heavily occluded with mineral crystallites. The ultrastructure of the complex hypermineralized layer in non-carious cervical sclerotic lesions varies. So, grinding or using more potent acids to remove the hypermineralized surface layers is a possible strategy to improve micromechanical retention in sclerotic dentine [19].

Until 2007, the most widely used system for clinical evaluation of restorations was the USPHS (United States Public Health Service) [20,21]. In 2007, the FDI World Dental Federation approved a new system of clinical criteria due to the low sensitivity of USPHS criteria in short-term clinical trials. From 2007 until today, this system has undergone changes and improvements, and the last one, published in 2010, has been used in the present clinical study [16,22]. Those criteria and the grading system were approved by the General Assembly in 2008 as “standard criteria” that should be applied when restorative materials and/or operative techniques are to be clinically investigated [22].

The null hypothesis of the study was partially rejected. According to our findings, the three bonding techniques provided high retention rates, ranging between 96.6% and 100% at the 18-month assessment. These values fall into the American Dental Association (ADA) requirement for full acceptance in clinical use, which must be higher than 90% [23]. Concerning the retention rates after 24 months of oral service, an average of 9.1% retention loss for the three bonding methods was achieved. The value is close to 10%, which has been reported in a meta-analysis study for 3 years of observation time [24].

Over the last two decades, many randomized clinical studies, systematic reviews, and meta-analysis investigations have been conducted to determine the loss percentage of resin composite restorations performed on non-caries cervical substrates [15,25,26,27,28]. However, the absence of standardization in clinical trial design leads to an extremely wide range of reported rates. Therefore, the outcomes related to the comparative assessment of different categories of adhesive systems tested under the same study may be considered a more reliable evaluation approach.

The results of the present clinical study did not show any statistically significant difference in terms of retention among the restorations placed with the self-etch, total-etch, and selective enamel etch and self-etch bonding methods (86.8%, 92.26%, and 93.63%, respectively). Thus, we can state that the type of bonding system does not influence the retention performance of the restorations over a mid-term period of 24 months.

Notably, the conclusions of systematic reviews that examined the risk of restoration loss using adhesives that belong to different adhesive strategies are controversial. Some studies revealed comparable outcomes among different bonding types/techniques for long-lasting post-operation periods [29,30,31,32], whereas others did not confirm such an effect [24,33,34,35]. Multiple variables of the adhesive systems related to the composition, application steps, acidity, and placement process cause the results’ heterogeneity. However, we should highlight that the studies that revealed the association of the adhesive type with the retention values state that the total-etch technique leads to a lower risk of restoration loss relative to the self-etch technique [33,34,35].

The obvious disagreement between these studies and our outcomes seems reasoned because meta-analysis studies include a wide range of adhesive types, commercial brands, and techniques, whereas, in our study, the used agents are produced by the same manufacturer and have a very similar composition. Concerning the impact of selective enamel etching, the literature certifies that it does not offer an advantage over self-etch [3,33].

Regarding the occurrence of caries at the margins of restorations, no statistical comparison was carried out among the three techniques because no case of caries incidence was detected during the 2-year evaluation period. The absence of caries is a repeated finding in most of the relevant clinical trials [6,11,36,37]. Even after eight years, Mahn et al. (2015) reported that the prevalence was nearly zero [34]. It is interesting to point out that in our study, perfect agreement (kappa = 1) was obtained between the two evaluators for retention and caries.

As a function of time, the restorations shifted gradually from the clinically perfect to an acceptable situation in terms of marginal adaptation. The relative decrease in the number of restorations with perfect marginal integrity was estimated at 17.9% per month. Imperfections along their margins were present in most of the restorations, regardless of the three bonding procedures applied. Specifically, after 24 months post-operation, the clinically perfect restorations were 26.9% for the total-etch technique, 16% for self-etch, and 25.9% for selective enamel etch and self-etch. Although the performance of self-etch adhesion was recorded as inferior compared with the two techniques employed with enamel etching, these differences were not statistically significant. However, it may indicate enhanced interlocking of the resin composite with etched enamel tissue. Indeed, in vitro studies have shown that higher bond strengths are provided by universal adhesives when the bonding application follows enamel etching [38].

All the non-perfect restorations were assigned as clinically acceptable because only minor local defects (less than 150 μm and 250 μm) were present. In addition, according to the current trial’s outcomes, the three adhesive techniques can yield equal quality of marginal adaptation, providing 100% satisfied marginal sites (perfect and acceptable grades 2 and 3).

Generally, marginal defects are usually detected beyond the beveled margins onto uncut enamel. According to in vitro studies, the inability of the self-adhesive systems to sufficiently etch the enamel, especially the uncut parts, leads to the creation of a bond that is more prone to degradation [39,40].

Nevertheless, under the conditions of the recent trial, such a difference in performance between total-etch and self-etch bonding procedures was not observed. A possible explanation may be that the less favorable adhesion is expressed as very small marginal defects, which are not easily noticeable under clinical examination in the post-treatment period of the study. This finding agrees with the conclusion of Josic et al. (2021) that self-etch and total-etch bonding strategies provide restorations with comparable marginal outcomes up to a 3-year follow-up [35], but it is in contrast with two other systematic reviews/meta-analyses that showed worse marginal performance in restorations with the use of self-etch relative to total-etch techniques [32,34]. Furthermore, Szesz et al. (2016) found that selective enamel etching provided better marginal quality than self-etching [3].

The patient-related parameters (gender, oral health attitudes, number of cervical abraded lesions), as single factors, did not interact with the changes in marginal adaptation in all groups of aged restorations.

According to the clinical evaluation, 3.12% of the restorations applied with the total-etch method, 26.11% with self-etch, and 21.64% with selective enamel etch and self-etch presented marginal staining, which was judged as mild. So, all the restorations are characterized as clinically acceptable for up to 2-year oral function. Although the discoloration values assigned fall into the range of mean values reported in systematic studies [24,34], direct comparison may be unreliable because different categories of restorative materials—resin composites and glass-ionomers—were analyzed all together in these studies.

The regression analysis revealed that when the self-etch agent is used even before enamel etching (methods B and C), the probability of marginal discoloration through time will be statistically higher than with the total-etch technique. Under the consideration that a similar correlation was not determined for marginal adaptation, we can claim that marginal imperfections may not be the strongest contributor to the marginal discoloration phenomenon [41,42]. Compared with total-etch techniques, the different enamel etching patterns produced by the self-etch adhesive might be attributed to the marginal discoloration outcomes. Irrespective of the etching pattern produced by the various self-etch systems, all their sub-categories provide a very shallow enamel etching with fewer micro-porosities for resin infiltration and weaker bond strength [6,43,44]. The particular quality of the interface/bond may favor the infiltration of stains and/or bacterial biofilm, causing margin pigmentation.

An in vitro study reported some moderate correlation between marginal staining and bond strength values after 6 months of water aging [45]. However, it is notable that, according to the current study’s findings, selective enamel etching, which aims to improve the interface, reduced the possibility of marginal discoloration relative to the total-etch system. We should record the discoloration separately along the enamel and dentin margins during the examination to ensure this hypothesis. If the stains are mainly absorbed in the dentin margins, the selective enamel etching may not have a remarkable impact on the total marginal discoloration, even in the case of reduced discoloration at the enamel part.

Most systematic studies have mentioned the superiority of the total-etch technique to the self-etch [24,29,32,34,46,47], whereas only Josic et al. (2021) reported no difference [35].

## 5. Conclusions

This randomized split-mouth controlled clinical trial compared the 2-year clinical performance of resin composite restorations placed at NCCLs with one-step self-etch, total-etch, and selective enamel etch and self-etch adhesive techniques. After 2 years of oral service, the three adhesive strategies provided restorations with no significant differences in retention rate or marginal adaptation. Through time, marginal adaptation and staining were becoming worse, although all the restorations were assessed as clinically acceptable after 2 years. None of the restorations presented a caries occurrence.

## Figures and Tables

**Table 1:** Materials used.

Material	Company	Type	Composition	Instructions
AdheSE One F	IvoclarVivadent, Schaan, Liechtenstein	One step, self-etch	Dimethacrylate, phosphonic acid acrylate, initiators and stabilizers in an aqueous solution HEMA, dimethacrylate, silicon dioxide, initiators, and stabilizers.	Brush onto the surface for >30 s; disperse excess with a strong stream of air; light cure for 10 s.
ExciTE F	IvoclarVivadent, Schaan, Liechtenstein	One step, total-etch	HEMA, dimethacrylates, phosphonic acid acrylate, highly dispersed silicon dioxide, initiators, and stabilizers in an alcohol solution.	Saturate enamel and dentine with a generous amount of the agent using the applicator; agitate the adhesive onto dentin surface for at least 10 s with a gentle stream; light cure for 10 s.
Tetric EvoCeram	IvoclarVivadent, Schaan, Liechtenstein		75–76% w, Barium glass, ytterbium trifluoride, mixed oxide, prepolymer (82–83%) 17–18% w organic matrix.	
N-etch	IvoclarVivadent, Schaan, Liechtenstein		37% phosphoric acid.	

**Table 2:** The distribution of teeth per method.

Teeth Group	Method A	Method B	Method C
Upper premolars	10	9	10
Lower premolars	9	10	9
Upper canines	7	7	6
Lower canines	4	4	5
Upper Incisors	2	2	2

**Table 3:** Descriptive statistics of patient-related parameters.

	Male *n* (%)	Female *n* (%)	Total *n* (%)	*p*-Value
Brushing frequency				0.386 *
>twice a day	9 (69.2)	16 (84.2)	25 (78.1)	
Once a day	3 (23.1)	3 (15.8)	6 (18.8)	
2–3 times per week	1 (7.7)	0 (0.0)	1 (3.1)	
Frequency of dental visits				0.185 *
1–2 times per year	2 (15.4)	7 (36.8)	9 (28.1)	
Whenever a problem exists	11 (84.6)	12 (63.2)	23 (71.9)	
	**Median**	**Median**	**Median**	***p*** **-Value**
Age	57 (54–65)	60 (50–72)	59.5 (51–65)	0.577 **
Number of teeth with cervical abraded surfaces	11	11	11	0.513 **

* Chi square, ** Mann-Whitney.

**Table 4:** Retention rates at baseline, 6-, 12-, 18-, and 24-months post-operation.

	Method A *n* (%)	Method B *n* (%)	Method C *n*(%)	*p* Value *
baseline				
Yes	32 (100)	32 (100)	32 (100)	
No	0 (0.0)	0 (0.0)	0 (0.0)	
6 months				0.37
Yes	32 (100)	31 (100)	31 (96.9)	
No	0 (0.0)	0 (0.0)	1 (3.1)	
12 months				0.382
Yes	29 (96.7)	28 (100)	29 (100)	
No	1 (3.3)	0 (0.0)	0 (0.0)	
18 months				0.604
Yes	28 (96.6)	27 (96.4)	28 (100)	
No	1 (3.4)	1 (3.6)	0 (0.0)	
24 months				0.593
Yes	26 (96.3)	25 (96.2)	27 (100)	
No	1 (3.7)	1 (3.8)	0 (0.0)	

* Chi-square.

**Table 5:** Marginal adaptation at baseline, 6-, 12-, 18-, and 24-months post-operation.

	Method A *n* (%)	Method B *n* (%)	Method C *n* (%)	*p* Value *
(grades 1–5) Baseline				0.77
(1) Perfect	31 (96.9)	31 (96.9)	30 (93.8)	
(2) Marginal gap < 150 μm	1 (3.1)	1 (3.1)	2 (6.3)	
6 months				0.448
(1) Perfect	22 (68.8)	16 (51.6)	20 (64.5)	
(2) Marginal gap < 150 μm	10 (31.3)	14 (45.2)	11 (35.5)	
(3) Marginal gap < 250 μm	0 (0.0)	1 (3.2)	0 (0.0)	
12 months				0.695
(1) Perfect	13 (44.8)	11 (39.3)	13 (44.8)	
(2) Marginal gap < 150 μm	16 (55.2)	16 (57.1)	16 (55.2)	
(3) Marginal gap < 250 μm	0 (0.0)	1 (3.6)	0 (0.0)	
18 months				0.532
(1) Perfect	9 (32.1)	6 (22.2)	10 (35.7)	
(2) Marginal gap < 150 μm	19 (67.9)	20 (74.1)	18 (64.3)	
(3) Marginal gap < 250 μm	0 (0.0)	1 (3.7)	0 (0.0)	
24 months				0.528
(1) Perfect	7 (26.9)	4 (16.0)	7 (25.9)	
(2) Marginal gap < 150 μm	18 (69.2)	20 (80.0)	20 (74.1)	
(3) Marginal gap < 250 μm	0 (3.8)	1 (4.0)	0 (0.0)	

* Chi-square.

**Table 6:** Marginal discoloration at baseline, 6-, 12-, 18-, and 24-months post-operation.

	Method A *n* (%)	Method B *n* (%)	Method C *n* (%)	*p* Value *
(grades 1–5) Baseline				
(1) No	32 (100.0)	32 (100.0)	32 (100.0)	-
6 months				0.352
(1) No	31 (96.9)	29 (93.5)	31 (100.0)	
(2) Minor	1 (3.1)	2 (6.5)	0 (0.0)	
12 months				0.147
(1) No	29 (100.0)	25 (89.3)	28 (96.6)	
(2) Minor	0 (0.0)	3 (10.7)	1 (3.4)	
18 months				0.252
(1) No	28 (100.0)	24 (88.9)	24 (85.7)	
(2) Minor	0 (0.0)	3 (11.1)	3 (10.7)	
(3) Moderate	0 (0.0)	0 (0.0)	1 (3.6)	
24 months				0.067
(1) No	26 (100.0)	19 (76.0)	21 (77.8)	
(2) Minor	0 (0.0)	6 (24.0)	5 (18.5)	
(3) Moderate	0 (0.0)	0 (0.0)	1 (3.7)	

* Chi-square.

**Table 7:** Univariate and multivariate logistic analysis of the likelihood of marginal adaptation (dependent variable, yes/no). Results of the final random effects logistic regression model.

Univariate Logistic Analysis				Multivariate Logistic Analysis		
	Odds Ratio	95% C.I	*p*-Value *	Odds Ratio	95% C.I	*p*-Value *
Time (months)	0.821	(0.798, 0.844)	<0.001	0.818	(0.795, 0.841)	<0.001
Age (years)	0.993	(0.972, 1.015)	0.539	1.003	(0.969, 1.038)	0.868
Gender						
Male	1			1		
Female	1.227	(0.749, 2.012)	0.417	1.308	(0.587, 2.915)	0.512
Method						
A	1			1		
B	0.779	(0.552, 1.097)	0.153	0.661	(0.429, 1.017)	0.059
C	0.900	(0.641, 1.262)	0.540	0.878	(0.574, 1.344)	0.549
Frequency of Brushing (2+ times/day)				ND **	ND	ND
No	1					
Yes	0.726	(0.406, 1.297)	0.279			
Frequency of visit at dentist				ND	ND	ND
1–2 times/year	1					
When a problem exists	0.744	(0.439, 1.261)	0.272			
Number of NCCL	0.979	(0.943, 1.017)	0.268	ND	ND	ND
Shape				ND	ND	ND
Curve	1					
Wedge	1.131	(0.742, 1.726)	0.567			

* Wald (Wald test), ** ND = Non-determined.

## Data Availability

No new data were created or analyzed in this study. Data sharing is not applicable to this article.

## References

[1] Perdigão J., Araujo E., Ramos R.Q., Gomes G., Pizzolotto L. (2021). Adhesive dentistry: Current concepts and clinical considerations. J. Esthet. Restor. Dent..

[2] Scotti N., Bergantin E., Giovannini R., Delbosco L., Breschi L., Migliaretti G., Pasqualini D., Berutti E. (2015). Influence of multi-step etch-and-rinse versus self-etch adhesive systems on the post-operative sensitivity in medium-depth carious lesions: An in vivo study. Am. J. Dent..

[3] Szesz A., Parreiras S., Reis A., Loguercio A. (2016). Selective enamel etching in cervical lesions for self-etch adhesives: A systematic review and meta-analysis. J. Dent..

[4] Ozel E., Say E., Yurdaguven H., Soyman M. (2010). One-year clinical evaluation of a two-step self-etch adhesive with and without additional enamel etching technique in cervical lesions. Aust. Dent. J..

[5] Pena C., Rodrigues J., Ely C., Giannini M., Reis A. (2016). Two-year Randomized Clinical Trial of Self-etching Adhesives and Selective Enamel Etching. Oper. Dent..

[6] Peumans M., De Munck J., Van Landuyt K., Poitevin A., Lambrechts P., Van Meerbeek B. (2010). Eight-year clinical evaluation of a 2-step self-etch adhesive with and without selective enamel etching. Dent. Mater..

[7] Van Meerbeek B., Yoshihara K., Yoshida Y., Mine A., De Munck J., Van Landuyt K.L. (2011). State of the art of self-etch adhesives. Dent. Mater..

[8] Dreweck F.D.S., Zarpellon D., Wambier L.M., Loguercio A.D., Reis A., Gomes O.M.M. (2021). Is There Evidence that Three-step Etch-and-Rinse Adhesives Have Better Retention Rates than One-step Self-etch Adhesives in Noncarious Cervical Lesions? A Systematic Review and Meta-Analysis. J. Adhes. Dent..

[9] Abdalla A.I., El Sayed H.Y. (2008). Clinical evaluation of a self-etch adhesive in non-carious cervical lesions. Am. J. Dent..

[10] Ermis R.B., Van Landuyt K.L., Cardoso M.V., De Munck J., Van Meerbeek B., Peumans M. (2012). Clinical effectiveness of a one-step self-etch adhesive in non-carious cervical lesions at 2 years. Clin. Oral Investig..

[11] Moretto S., Russo E., Carvalho R., De Munck J., Van Landuyt K., Peumans M., Van Meerbeek B., Cardoso M. (2013). 3-year clinical effectiveness of one-step adhesives in non-carious cervical lesions. J. Dent..

[12] van Dijken J.W., Pallesen U. (2012). A 7-year randomized prospective study of a one-step self-etching adhesive in non-carious cervical lesions. The effect of curing modes and restorative material. J. Dent..

[13] Peumans M., De Munck J., Van Landuyt K., Van Meerbeek B. (2015). Thirteen-year randomized controlled clinical trial of a two-step self-etch adhesive in non-carious cervical lesions. Dent. Mater..

[14] van Dijken J.W., Sunnegårdh-Grönberg K., Lindberg A. (2007). Clinical long-term retention of etch-and-rinse and self-etch adhesive systems in non-carious cervical lesions: A 13 years evaluation. Dent. Mater..

[15] de Siqueira F.S.F., Wendlinger M., Araújo L.C.R., Moreira P.H.d.A., Cardenas A.F.M., Carvalho T.S., Reis A., Loguercio A.D. (2023). Bonding performance of universal adhesives to eroded dentine: A 6-year evaluation. J. Dent..

[16] Hickel R., Peschke A., Tyas M., Mjör I., Bayne S., Peters M., Hiller K.-A., Randall R., Vanherle G., Heintze S.D. (2010). FDI World Dental Federation—Clinical Criteria for the Evaluation of Direct and Indirect Restorations. Update and Clinical Examples. J. Adhes. Dent..

[17] Caneppele T.M.F., Meirelles L.C.F., Rocha R.S., Gonçalves L.L., Ávila D.M.S., Gonçalves S.E.d.P., Bresciani E. (2020). A 2-year clinical evaluation of direct and semi-direct resin composite restorations in non-carious cervical lesions: A randomized clinical study. Clin. Oral Investig..

[18] Tay F.R., Pashley D.H. (2004). Resin bonding to cervical sclerotic dentin: A review. J. Dent..

[19] Hopewell S., Loudon K., Clarke M.J., Oxman A.D., Dickersin K. (2009). Publication bias in clinical trials due to statistical significance or direction of trial results. Cochrane Database Syst. Rev..

[20] Cvar J.F., Ryge G. (2006). Reprint of Criteria for the clinical evaluation of dental restorative materials. Clin. Oral Investig..

[21] Bayne S.C., Schmalz G. (2005). Reprinting the classic article on USPHS evaluation methods for measuring the clinical research performance of restorative materials. Clin. Oral Investig..

[22] Hickel R., Roulet J.F., Bayne S., Heintze S.D., Mjor I.A., Peters M., Rousson V., Randall R., Schmalz G., Tyas M. (2007). Recommendations for conducting controlled clinical studies of dental restorative materials. Science Committee Project 2/98--FDI World Dental Federation study design (Part I) and criteria for evaluation (Part II) of direct and indirect restorations including onlays and partial crowns. J. Adhes. Dent..

[23] American Dental Association—Council on Scientific Affairs (2001). Dentin and Enamel Adhesive Materials.

[24] Heintze S.D., Ruffieux C., Rousson V. (2010). Clinical performance of cervical restorations—A meta-analysis. Dent. Mater..

[25] Mathias-Santamaria I.F., Santamaria M.P., Silveira C.A., Martinho F.C., de Melo M.A.S., De Marco A.C., Augusto M.G., de Andrade G.S., Roulet J.-F., Bresciani E. (2023). Evaluation of a novel restorative protocol to treat non-carious cervical lesion associated with gingival recession: A 2-year follow-up randomized clinical trial. Clin. Oral Investig..

[26] Ñaupari-Villasante R., Matos T.P., de Albuquerque E.G., Warol F., Tardem C., Calazans F.S., Poubel L.A., Reis A., Barceleiro M.O., Loguercio A.D. (2023). Five-year clinical evaluation of universal adhesive applied following different bonding techniques: A randomized multicenter clinical trial. Dent. Mater..

[27] Matos T., Hanzen T., Almeida R., Tardem C., Bandeca M., Barceleiro M., Loguercio A., Reis A. (2022). Five-year Randomized Clinical Trial on the Performance of Two Etch-and-rinse Adhesives in Noncarious Cervical Lesions. Oper. Dent..

[28] Follak A.C., Ilha B.D., Oling J., Savian T., Rocha R.d.O., Soares F.Z.M. (2021). Clinical behavior of universal adhesives in non-carious cervical lesions: A randomized clinical trial. J. Dent..

[29] Schroeder M., Correa I.C., Bauer J., Loguercio A.D., Reis A. (2017). Influence of adhesive strategy on clinical parameters in cervical restorations: A systematic review and meta-analysis. J. Dent..

[30] Krithikadatta J. (2010). Clinical effectiveness of contemporary dentin bonding agents. J. Conserv. Dent..

[31] Santos M.J.M.C., Ari N., Steele S., Costella J., Banting D. (2014). Retention of tooth-colored restorations in non-carious cervical lesions—A systematic review. Clin. Oral Investig..

[32] Fang K., Chen K., Shi M., Wang L. (2023). Effect of different adhesive systems on dental defects and sensitivity to teeth in composite resin restoration: A systematic review and meta-analysis. Clin. Oral Investig..

[33] Peumans M., De Munck J., Mine A., Van Meerbeek B. (2014). Clinical effectiveness of contemporary adhesives for the restoration of non-carious cervical lesions. A systematic review. Dent. Mater..

[34] Mahn E., Rousson V., Heintze S. (2015). Meta-Analysis of the Influence of Bonding Parameters on the Clinical Outcome of Tooth-colored Cervical Restorations. J. Adhes Dent.

[35] Josic U., Maravic T., Mazzitelli C., Radovic I., Jacimovic J., del Bianco F., Florenzano F., Breschi L., Mazzoni A. (2021). Is clinical behavior of composite restorations placed in non-carious cervical lesions influenced by the application mode of universal adhesives? A systematic review and meta-analysis. Dent. Mater..

[36] Peumans M.J., Munck K., Van Landuyt P., Lambrechts B., Van Meerbeek B. (2005). Three-year clinical effectiveness of a two-step self-etch adhesive in cervical lesions. Eur. J. Oral Sci..

[37] Van Landuyt K.L., Peumans M., De Munck J., Cardoso M.V., Ermis B., Van Meerbeek B. (2011). Three-year clinical performance of a HEMA-free one-step self-etch adhesive in non-carious cervical lesions. Eur. J. Oral Sci..

[38] Cuevas-Suarez C.E., Rosa W.L.d.O.d., Lund R.G., da Silva A.F., Piva E. (2019). Bonding Performance of Universal Adhesives: An Updated Systematic Review and Meta-Analysis. J. Adhes. Dent..

[39] Frankenberger R., Tay F.R. (2005). Self-etch vs etch-and-rinse adhesives: Effect of thermo-mechanical fatigue loading on marginal quality of bonded resin composite restorations. Dent. Mater..

[40] Moura S.K., Reis A., Pelizzaro A., Dal-Bianco K., Loguercio A.D., Arana-Chavez V.E. (2009). Bond strength and morphology of enamel using self-etching adhesive systems with different acidities. J. Appl. Oral Sci..

[41] Van Landuyt K., Peumans M., Fieuws S., De Munck J., Cardoso M., Ermis R., Lambrechts P., Van Meerbeek B. (2008). A randomized controlled clinical trial of a HEMA-free all-in-one adhesive in non-carious cervical lesions at 1 year. J. Dent..

[42] Turkun S.L. (2003). Clinical evaluation of a self-etching and a one-bottle adhesive system at two years. J. Dent..

[43] Goracci C., Rengo C., Eusepi L., Juloski J., Vichi A., Ferrari M. (2013). Influence of selective enamel etching on the bonding effectiveness of a new “all-in-one” adhesive. Am. J. Dent..

[44] Mine A., De Munck J., Cardoso M.V., Van Landuyt K.L., Poitevin A., Kuboki T., Yoshida Y., Suzuki K., Lambrechts P., Van Meerbeek B. (2009). Bonding effectiveness of two contemporary self-etch adhesives to enamel and dentin. J. Dent..

[45] Heintze S.D., Thunpithayakul C., Armstrong S.R., Rousson V. (2011). Correlation between microtensile bond strength data and clinical outcome of Class V restorations. Dent. Mater..

[46] Peumans M., Vandormael S., De Coster I., De Munck J., Van Meerbeek B. (2023). Three-year Clinical Performance of a Universal Adhesive in Non-Carious Cervical Lesions. J Adhes Dent..

[47] de Oliveira R.P., de Paula B.L.F., de Melo Alencar C., Alves E.B., Silva C.M. (2023). A randomized clinical study of the performance of self-etching adhesives containing HEMA and 10-MDP on non-carious cervical lesions: A 2-year follow-up study. J. Dent..

